# Treatment Adherence with Early Prescription of Long-Acting Injectable Antipsychotics in Recent-Onset Schizophrenia

**DOI:** 10.1155/2012/368687

**Published:** 2012-04-03

**Authors:** Annie Viala, Françoise Cornic, Marie-Noëlle Vacheron

**Affiliations:** SM 13, Centre Hospitalier Sainte-Anne, 1 Rue Cabanis, 75014 Paris, France

## Abstract

Although response to treatment for the first episode of schizophrenia is generally favourable, nonadherence with the treatment is the first cause of relapse and rehospitalisation within the next few years. Long-acting injectable antipsychotics (LAIAs) combine the advantages of the newer antipsychotics and the long-acting formulation. The evaluation concerns 25 schizophrenic patients hospitalised for the first time, treated with risperidone long-acting injectable (RLAI) associated with reintegration methods, and followed up for at least 18 months. Clinical observation was completed using Clinical Global Impression (CGI) scale and Global Assessment of Functioning (GAF). Clinical improvement was coupled with a good reintegration rate, very few relapse, or rehospitalisation. Bimonthly injection combined with psychosocial methods improved interactive followup, and therefore patients' compliance with the treatment. Treating with LAIA as early as possible, from the first episode if possible, can reduce relapse, number and duration of rehospitalisation, and cognitive symptoms and improve the quality of life and prognosis.

## 1. Introduction

Schizophrenia remains a chronic disease concerning about 1% of the general population in the world; although response to treatment in the early phases of evolution is generally favourable, it is estimated that rates of adherence to treatment 1 year after discharge from hospital are only about 50%, and 75% in the first two years of treatment [1, 2].

The causes are multifactorial: denial of disease, side effects of medication, cognitive impairment, comorbidity especially substance abuse [3] and also doctor- patient relationship [4].

Relapses and rehospitalisations worsen the prognosis of patients with schizophrenia, impact both patients and families'insertion and quality of life, and increase direct and indirect health costs [5, 6]. Treatment with LAIAs, which encourage adherence (fewer side-effects, stabilization of drug levels), can prevent the risk of interruption of treatment which is the main cause of relapse and rehospitalisation. This type of treatment, used as early as possible, since first-episode schizophrenia, may improve the long-term prognosis [7], particularly when associated with reintegration methods [8] and interactive followup using interest of monthly or bimonthly injection.

The aim of our study is to present the followup in “real life” of 25 patients, hospitalised for the first time, treated with RLAI associated with reintegration methods and multidisciplinary followup, for at least 18 months, in order to evaluate the impact: 1/on relapse and rehospitalisation rate; 2/on treatment adherence.

## 2. Materials and Methods

### 2.1. Study Design

The data presented here derive from a naturalistic prospective study of 120 patients treated with Risperidone Long Acting Injectable (RLAI) which was currently the only atypical long-acting injectable antipsychotic available at that time in France, associated with rehabilitation methods, and followed up for at least 18 months [9]: among them, 25 patients were hospitalised for the first time, and therefore younger in age and illness duration.

It was an epidemiological, observational, noninterventional study in usual-care settings, in which patients were treated with flexible dose of RLAI, without randomization nor controlled trial. All patients were aware of the aim of the study and had given oral informed consent.

The aim of our study was to investigate the outcome of the illness according to a better compliance with RLAI integrated in a psychosocial treatment programme, notably the number and duration of hospitalisations, and also the possibility of familial and socioprofessional reintegration.

### 2.2. Inclusion and Exclusion Criteria

All patients were men or women of minimally 18 years of age, recruited consecutively in the same catchman urban area corresponding to their district of psychiatric healthcare in Paris (all of them were residents of the 14th district of Paris). All of them were psychotics according to DSM IVR criteria for schizophrenia [10].

Exclusion criteria were serious medical condition, history of malignant syndrome, pregnant or breast-feeding female, history of clozapine treatment, known allergies, hypersensitivity or intolerance to risperidone, and patients who had not given informed consent.

### 2.3. Assessments

Patients were assessed at baseline and after 6, 12, and 18 months for adherence, efficacy, RLAI dosage, number and duration of hospitalisation, social functionality, and reintegration (work, studies, apartment).

Clinical observation was completed using Clinical Global Impression scale (CGI) [11] representative of clinical improvement, and Global Assessment of Functioning (GAF) [12] representative of functional improvement, whose results are statistically significant.

### 2.4. Treatment

All patients were treated with risperidone per os, with doses ranging from 4 mg to 8 mg before changing to RLAI. The first injection was given prior to discharge, and the oral treatment was continued for 3 or 4 weeks.

The following injections were given at the out-patient center, or in the hospital depending on where each patient was consulted, and on their need to maintain a more controlled therapeutic regimen. The nurses supervised administration of the treatment and could call them when they missed time of injection. The starting dose was calculated according to the patient's acute state and comorbidity, especially substance abuse (alcohol, cannabis). The initially prescribed dosage changed over time, according to the evolution, and to the physician.

### 2.5. Reintegration Measures

Day hospital, part-time therapy center, sheltered housing, protected employment center, and also dietetic education, physical exercise were used; each patient was cared for by two nurses and a social worker; these measures were adapted to each patient's needs, using bimonthly injection to consult their physician and/or a nurse.

### 2.6. Statistical Analyses

Descriptive statistics (mean standard deviation) were generated for quantitative data for all patients. For statistical analysis of the CGI and GAF data, subgroups were created with those patients who completed the survey after 18 months. Statistical analysis of hospitalisation data was performed for the subgroup of patients who had received treatment during the preceding 18 months, who completed the 18-month followup, and who had at least one hospitalisation during these two time periods. Quantitative data was first tested for normality using the Kolmogorov-Smirnov test, followed by analysis for statistical significance using either the Wilcoxon signed rank test or the paired Student's *t*-test, as applicable. Qualitative data was subjected to McNemar's test. Bowker's test for symmetry was used to analyse dosage change over time.

## 3. Results

### 3.1. Sociodemographic Data and Clinical Characteristics

The patient population comprised 14 men and 11 women, the mean age was 30,08 ± 7,54 years, the mean duration of illness 6,84 ± 5,45 years; all of them were hospitalised for the first time, in acute or very acute state (positive symptoms: 20 patients, negative symptoms: 5 patients); the most common diagnosis was paranoid schizophrenia (13 patients), followed by schizoaffective disorder (5 patients), disorganised schizophrenia (3 patients), undifferentiated schizophrenia (2 patients), and acute psychotic disorder (2 patients).

### 3.2. Previous Treatment

17 of 25 patients (64%) were not taking any treatment before being hospitalized; only 3 of them were naïve of all treatments. 8 of 25 were treated with Olanzapine (4 patients), Haloperidol (3 patients), Pipotiazine (1 patient). 

None of them had previously received Risperidone. 

Reasons for prescription were lack of efficacy or compliance (16 patients), lack of tolerability (2 patients), prescriber's choice (4 patients), and comorbidity such as substance abuse.

### 3.3. Dosage, Tolerability, Discontinuation Rate

RLAI dosage changed over time: 24 received a starting dosage of 50 mg, according to their very acute state and coexisting alcohol dependence and substance abuse (especially cannabis use), and also our normal clinical practice; 1 received a starting dose of 37,5 mg. The dosage has been decreased during the course of the survey. Most patients (16 patients) went on 50 mg, some of them went on 37,5 mg or 25 mg, 5 of them were treated with 25 mg at M18, end of our study.

Five patients interrupted their treatment (including 3 quickly after initiation) because of illness denial, weight gain, or partial efficacy.

### 3.4. Socioprofessional Evolution

After RLAI instauration, 19 patients could live in their own apartment, 1 in sheltered housing; 11 could restart work or find a job (100%: 5 patients, 50%: 4 patients, professional training: 2 patients), 3 patients could restart studies. Other patients had activities in day hospital or part-time therapy center, some of them needing some more time to attend a protected employment center. All of them improved their quality of life over time, with more possibilities to meet friends and family, to live a more stable and independent life, outside the psychiatric hospital.

### 3.5. Clinical Efficacy CGI

Evolution of the CGI score is representative of clinical improvement: the level of severity of the illness (CGI-S) decreased from 5.44 (range 4–6, SD ± 0.58) on day 0 to 3.14 (range 1–5, SD ± 1.21) at M18. This result is statistically significant (*P* < 0.0001). CGI-I score showed that at M18, 3 patients were very greatly improved, 3 greatly improved, and 1 slightly improved.

### 3.6. Functional Improvement GAF

Evolution on the GAF scale is representative of functional improvement: the score increased from 36.16 (range 11–70, SD ± 13.55) on day 0 to 75.71 (range 65–85, SD ± 7.32) at M18. This result is statistically significant (*P* < 0.0001). The largest improvement was observed during the first 6 months of treatment, during which the mean GAF score increased by 20 and then more slowly until M18.

### 3.7. Hospitalisation Rate and Duration

Only 4 patients relapsed (16%), and only once: 3 patients between D0 and M6 (mean duration of hospitalisation: 21 days), and 1 during M6 and M12 (mean duration of hospitalisation: 7 days); no patient relapsed between M12 and M18. These results, when compared with the whole cohort's results, show that fewer patients were hospitalised after RLAI therapy, with a short duration of hospitalisation. We cannot compare with their own rate of hospitalisation, because it is their first hospitalisation, but we can notice that their rate of hospitalisation is better when compared with those who had already been treated and hospitalised in the whole cohort (37% for the whole cohort versus 16% for the 25 recent onset patients).

## 4. Discussion

These results compared with those of the whole cohort (120 patients) are very consistent and very close to them: marked improvements in CGI and GAF scores, reduced rates and durations of hospitalisation. They are globally better, and we think that is due to the recent onset in this population (patients younger in age and duration of illness) and they convince us to begin RLAI treatment as soon as possible (CGI improvement from 5,6 to 3,6 for the whole cohort versus 5,4 to 3,1 for 25 recent onset patients; GAF improvement from 34,1 to 67,5 for the whole cohort versus 36,1 to 75,7 for the 25 recent onset patients).

We currently used starting dosage of 50 mg, according to the very acute state and comorbidity (especially alcohol and cannabis use) of our patients, but also to our normal clinical practice in front of this “very difficult-to-treat-population,” considering underdosing can contribute discontinuation when patients are severely ill [13, 14], and also lower plasma levels of Risperidone and 9OH risperidone at the beginning of the treatment [15, 16]

Clinical benefits may be linked with psychosocial programme associated with RLAI treatment from the beginning of the treatment, and multidisciplinary long-term followup (18 months in our study) in order to improve adherence with the medication.

Treatment plan including early warning signs developed for each patient, based on psychoeducation programme, family involvement in order to acquire and improve insight are regularly emphazised [1, 17–19].

Interest in functional outcome is based on subjective satisfaction with life, occupational and social functioning [18, 20], less and shorter inpatient stays, less long-term institutionalization, solitary living, and dependence on disability pension; greater frequency of outpatient visits, better information, improved access to supported employment, and rehabilitation should help the patients to agree with LAIA formulation [21, 22].

The importance of early intervention in schizophrenia treatment [23, 24] is based on acute clinical symptomatology (often positive symptoms), high risk of non- and partial adherence, risk of accruing morbidity and persistent deficits [25], and also high sensitivity to antipsychotics.

Treating as early as possible, from the first episode if possible, can reduce relapse, number and duration of hospitalisation, and also cognitive symptoms, illness worsening and suicide attempts [26], and thus improving the prognosis, and also direct and indirect cost [27–30].

Long-acting injectable antipsychotics combine the advantages of both the newer antipsychotics (efficacy, fewer extrapyramidal symptoms) and the long-acting formulation [31, 32]; they can reduce relapse through the increased medication adherence in patients with schizophrenia [33] and lower fluctuations in plasma concentrations.

Although response to treatment for the initial psychotic episode is generally favourable, most patients are unable to maintain this improvement [25, 34, 35]; poor adherence may be particularly common after a first episode of schizophrenia and may occur very early [22]: according to Coldham et al., 39% are not adherent and 20% inadequately adherent in the first year of treatment; non-adherence with medication is the first cause of relapse, it could be one of the most preventable, but it stays one of the most difficult to solve [1].

According to the neurodegenerative theory, relapse and recurrences make brain structures more neurotoxic: ventricular enlargement, cortical atrophy in brain, longer duration of illness, less effectiveness of the medication, deficient cognitive function, and prominent negative symptoms [23, 36].

Relapse predictors include medication discontinuation as a high risk [34]; relapse prevention is a major objective, and long-term treatment is indicated [37]. In recent publications concerning patients in the early phase of schizophrenia, two potentially risk factors for rehospitalisation are confirmed: short duration of first hospitalisation (shorter than two weeks) and early nonadherence to medication [38], even though periods of nonadherence are brief [35].

Long-acting injectable antipsychotics should not be considered by psychiatrists as a last resort in persistently ill patients; some recent studies show that patients, especially young patients, with a good insight, and sometimes a high study level, when informed on long-acting formulation, agree with them and prefer to receive their medication via a long-acting formulation rather than in tablet form [39, 40]. Psychiatrists' attitude towards LAIA should evolve [4, 41]. They should be recommended as a part of an integrated treatment plan, including multidisciplinary followup and treatment teams administering and supervising the medication [21], using interest of monthly or bimonthly injection in order not to forget treatment, and also to improving more frequent and regular contact between patients and healthcare professionals, interactive followup with nurse, psychiatrist, and also peers [14, 42].

A number of studies and pharmacoeconomic models have demonstrated that long-acting risperidone decreases direct healthcare costs largely by reducing the rates of relapse and hospitalisation [5, 43].

Relapse and hospitalisations are costly: direct medical costs in healthcare, indirect costs on the basis of productive activity (for the patient and family) [6, 29]. Even though adherence interventions in psychotic disorders have produced mixed results, possible cost benefits for the society and prevention of hospitalisation are necessary for patients [44] and a challenge for public health.

**Limitations**

No randomization, nor comparative group, the small number of patients are limitations to our study. However, results are encouraging when compared with those of the whole cohort, and the recent publications of the literature. They will need further investigations.

## 5. Conclusion

The treatment of schizophrenia is an ongoing challenge in psychiatry. The literature on long-acting injectable antipsychotics for early-phase schizophrenia, first episode, or recent onset, is limited, there are very few long-term data and guidelines are not yet available [38, 45, 46].

Using injectable long-acting antipsychotics as initial therapeutic treatment can reduce relapse rate and improve the prognosis and should be maintained in the long-term treatment of schizophrenia, using interactive, interdisciplinary followup, corresponding with psychosocial biological continuum, in order to progress from compliance to adherence: working with family and caregivers, information about the illness and its treatment, psychoeducation, bimonthly injection combined with nurse and psychiatrist assessment, telephone call and home visit when patient is late, and reintegration programme (housing, work, studies) may improve adherence and outcomes in these patients. An additional pertinent function is motivational enhancement fostering compliance and active participation in schizophrenia treatment plans for patients, relatives, and psychiatrists.
